# Dissecting the development of bovine testicular tissue using spatial transcriptomics

**DOI:** 10.1186/s40104-025-01340-4

**Published:** 2026-02-05

**Authors:** Haoyan Jin, Yuan Ma, Yaru Xie, Shunkai Yang, Xiaoxu Chen, Nana Wang, Lingkai Zhang, Yun Ma

**Affiliations:** 1 College of Animal Science and Technology, Ningxia University, Yinchuan, 750021 China; 2 Key Laboratory of Ruminant Molecular Cell Breeding, Ningxia Hui Autonomous Region, Yinchuan, 750021 China; 3 College of Animal Science and Technology, Northwest A&F University, No. 22 Xinong Road, Yangling, 712100 Shaanxi China

**Keywords:** Bovine, Spatial transcriptome, Spermatogenesis

## Abstract

**Background:**

Mammalian spermatogenesis is critical for the transmission of male genetic information, and single-cell sequencing technology can reveal its complex process. However, at present, there is no research on the dynamic transcription of bovine germ cell population.

**Results:**

In this study, we used Stereo-seq to construct a spatial transcription map of bovine testicular tissue at two ages. Four germ cell groups and five somatic cell groups were determined, and functional enrichment characterized their different biological functions and the differences between calves and adult bulls. At the same time, we also defined the subpopulations of cells and marker genes, then, clarified the communications between germ cells.

**Conclusion:**

Our study constructed a spatial transcription map of bovine testicular tissue for the first time, and systematically described the dynamic transcription changes during spermatogenesis. These data laid the foundation for the study of spermatogenesis in large mammals and elucidated the transcriptional dynamics underlying male germ cell development.

**Supplementary Information:**

The online version contains supplementary material available at 10.1186/s40104-025-01340-4.

## Introduction

A stable spermatogenic epithelial cycle is conducive to the continuous production of high-quality sperm, enabling spermatogonia to smoothly transition to spermatocytes and ultimately to spermatids, a process that occurs continuously in the seminiferous tubules [1]. Spermatogonia cells maintain stemness renewal and differentiation through the GDNF-PI3K/AKT-PLZF axis. Under the trigger of retinoic acid (RA), they differentiate into spermatocytes and enter the meiosis process, completing haploidization to form round spermatid. Ultimately, the acrosome vesicle covers the nucleus, proteins undergo replacement, and flagella are formed, releasing mature sperm [2–4].

This developmental process is accompanied by dynamic gene expression, and RNA sequencing (RNA-seq) is now capable of analyzing male germ cell populations after high-purity sorting [5, 6]. With the advancement of single-cell sequencing technology, testicular cell atlases have been constructed for mammals such as humans, mice, and pigs. Based on the characteristics of marker expression, germ cell populations have been identified. Researchers have simulated the process of spermatogenesis using pseudotime series methods, and the heterogeneity among germ cells is no longer difficult to trace [7–9].

However the developmental process within the seminiferous tubules is intricate, and it is challenging to characterize the interactions between cells and the microenvironment’s regulation of germ cells post-dissociation. Single-cell transcriptomics is not sufficiently precise to adequately demonstrate this phenomenon. The emergence of spatial transcriptomics technologies has the potential to address this critical gap [10]. Stereo-seq has been shown to exhibit superior resolution in comparison to other spatial sequencing technologies that capture mixed expression signals from multiple cells. This enhanced resolution is a result of Stereo-seq's ability to combine macro- and micro-level capabilities. Therefore, we employed Stereo-seq [11] to capture in situ transcriptional dynamics during spermatogenesis in the testis to investigate the heterogeneity of testis development in calves and adult bulls.

In this study, we performed spatial transcriptomic sequencing of testicular tissues from calves and adult bulls, mapping the spatial localization of germ cells and somatic cells in these two species. We proceeded to delineate the various germ cell subpopulations and somatic cell subpopulations. We then conducted developmental trajectory analysis for the germ cell subpopulations. A subsequent analysis of five germ cell markers in bovine testes was conducted, and the results were characterized using fluorescent microscopy. Furthermore, the study revealed biological functional heterogeneity in the developmental processes of spermatogonia cells and somatic cells between calves and adult cattle, and explored their interaction patterns. Our research is expected to develop new molecular markers for early selection and accelerate the breeding process of high-fertility bull lines. At the same time, the map provides a new perspective for understanding the biological mechanism of bovine spermatogenesis.

## Methods

### Collection of testis samples

Testicular tissue samples were collected from three 12-month-old bulls and four 1-month-old calves. For the two groups of tissues, we collected bovine testis tissue from the same side immediately after slaughter, and the feeding and management conditions of the cattle were the same. The freshly collected testicular tissues were immediately immersed in phosphate-buffered saline (PBS; G4202-100ML, Servicebio, Wuhan, China) supplemented with 5 × penicillin/streptomycin antibiotic and maintained at 0–4 °C during transport to the laboratory using cooling units. All procedures were approved by the Experimental Animal Manage Committee of Ningxia University and were carried out in accordance with the Guide for the Care and Use of Laboratory Animals (NXU-2023-098).

### Tissue embedding

Following transportation to the laboratory, the testicular specimens were carefully dissected to remove connective fascia. The central tissue blocks were isolated, sectioned into 5 mm^3^ fragment, and immediately embedded in optimal cutting temperature (OCT) compound (Tissue-Tek; 4583, Sakura Finetek, Tokyo, Japan). The tissue blocks form the same group were embedded in the same 1 cm^3^ embedding box, including four biological replicates before sexual maturity and three biological replicates after sexual maturity. Specimens were snap-frozen using dry ice and subsequently archived in −80 °C ultra-low temperature freezer.

The residual tissue fragments were immersed in Bouin’s fixative (BL-G016, Nanjing SenBeiJia Biological Technology Co., Ltd., China) for 12 h to complete histological fixation. After fixation, specimens underwent sequential dehydration through a graded ethanol series, cleared in xylene, and infiltrated with paraffin. Samples were stored long-term at 4 °C.

### Histology and immunostaining

Paraffin (WGHB-319213129, Servicebio, Wuhan, China) blocks were sectioned at a thickness of 5 μm per slice. For histological staining, sections were deparaffinized, rehydrated and co-stained with hematoxylin and eosin (G1120, Solarbio, Beijing, China). For immunofluorescence staining, we boiled the sections for 15 min after deparaffinized them using 1 × Tris-EDTA antigen repair solution (G1206, Servicebio) and cooled them to room temperature, washed with PBS, and blocked with 3% BSA for 1 h. Tissue sections were incubated overnight at 4 °C with primary antibodies, including mouse anti-UCHL1 (1:200; ab23, abcam, UK), rabbit anti-SCP3 (1:200; 23024-1-AP, Proteintech, Wuhan, China), rabbit anti-TNP1 (1:200; 17178-1-AP, Proteintech), rabbit anti-SLTM (1:200; 17889-1-AP, Proteintech), rabbit anti-DMRT1 (1:200; 14313-1-AP, Proteintech), rabbit anti-TKTL1 (1:200; bs-6284R; Bioss, Beijing, China), rabbit anti-YBX2 (1:200; bs-12271R, Bioss) and rabbit anti-AKAP4 (1:200; 24986-1-AP, Proteintech), the next day incubated 488-conjugated donkey anti-mouse and 594-conjugated donkey anti-rabbit (1:200; Thermo Fisher Scientific; USA) secondary antibodies. After the last PBS wash, the slice was sealed with anti-fluorescence sealer and then photographed with fluorescence microscope. All immunofluorescence results were compared against negative controls to rule out non-specific staining.

### Stereo-seq library construction

OCT-embedded tissues were cut into 10 μm frozen sections, which were affixed to Stereo-seq T vector microarrays (201ST114, BGI, Shenzhen, China), incubated at 37 °C for 4 min, followed by methanol fixation, H&E staining, image scanning, tissue permeabilization, reverse transcription, tissue removal, cDNA recovery and purification with the STOmics Stereo-seq transcription kit. Finally, DNA libraries were constructed using Stereo-seq library construction kit [11].

### Stereo-seq data processing and gene quantification

The Stereo-seq was performed by OE Biotech Co., Ltd. (Shanghai, China). All QC-qualified libraries were subjected to high-throughput sequencing on DNBSEQ-T7, and the raw data generated by sequencing were fastq sequences. The spatial transcriptome data and bright field microscope section images were processed using the BGI SAW (version5.5.4) software package [11]. The objective is to detect the captured region of testicular tissue in the chip and distinguish the reads enriched in each DNA nanoball (DNB) based on the CID (Coordinated Identifier) information. The next step is to obtain the quantitative information of genes at bin50 resolution by tissue segmentation. Then, the number of reads, number of genes, and MID (Molecular Identifier) in each spot at this resolution must be quantified, so that, the quality of the samples was assessed. A total of 1,847,996,007 reads were measured in calves, with 90.19% Q30, 90.61% Q30 for CID and 85.58% Q30 for MID; a total of 1,815,987,137 reads were measured in bulls, with 92.48% Q30, 90.0% Q30 for CID and 88.85% Q30 for MID, and the above results can be used for the following analysis. In this step, a bin size of 50 (50 × 50 spots) was used as the analytical unit for the annotation of Stereo-seq slides.

### Quantitative gene quality control and data preprocessing

Integration of STAR software, the reads obtained from sequencing were compared to the reference genome of *Bos taurus* (ARS-UCD2.0) for comparative analysis, and the expression matrix of gene-CID was generated for the analysis of relevant gene expression [12]. The number of clean reads for calves was 100,851,273 and the number of uniquely mapped reads was 7,887,865. For bulls, the number of clean reads was 780,747,785, and the uniquely mapped reads was 656,649,857. Based on the initial QC results of the SAW, the data were further processed using the Seurat software package, normalized using sctransform, and stored in the SCT matrix, after detecting the variance features. We used the publicly available pipeline SAW on https://github.com/STOmics/SAW [13, 14].

### Dimensionality reduction and clustering

Bovine testis expression profile matrix was binned into bin50, and the top 3,000 highly variable genes (HVGs) were screened using the FindVariableGenes function in the Seurat package. Subsequently, the expression profiles of these HVGs were linearly downscaled via principal component analysis (PCA). Finally, the results were visualized in 2D space by UMAP [15].

### Cell type mapping

After initial identification of cell clusters by unsupervised clustering based on similarity of gene expression, we compiled a list of celltype-specific marker genes from published literature and single-cell RNA sequencing databases (PanglaoDB, CellMarker) [16]. The cell population was then annotated by semi-supervised, and each cell cluster was re-annotated by accessing the average expression of typical markers, including 4 testicular germ cell clusters and 5 somatic cell clusters.

### Spatial characterization of marker genes

Marker genes for each cell population were identified using the FindAllMarker function (test.use = bimed) in the Seurat package, which detects genes differentially upregulated in one population compared to all others. The results were then visualized with the VInPlot and FeaturePlot functions.

### Differential genes and enrichment analysis

Differential gene screening was performed using the FindMarkers function (test.use = wilcox) in the Seurat package, and differential genes were screened according to the conditions of *P* value less than 0.05 and the number of differential loci greater than 1.5-fold, and GO and KEGG enrichment analyses of differentially significant genes were carried out by hypergeometric distribution test. As a result, we performed FDR correction and applied it to subsequent studies.

### Slingshot analysis of developmental trajectories

Testis cell developmental trajectory analysis was performed using the Slingshot package, trajectory inference was performed after converting the downscaled Seurat object into a SingleCellExperiment object, and downstream analyses including trajectory analyses of gene expression were performed with the tradeSeq package [17].

### Cellular communication analysis

In order to clarify the communication between various cell types, the gene expression matrix of each cell cluster was input into the Cellchat V2 package. The number of interactions, the intensity of interactions, and the overall network of interactions of each cell cluster were visualized [18].

## Results

### Construction of a spatial transcriptional map of bovine testis tissue

Prior to the shipment of the specimen for measurement, the integrity of the testicular tissue was ascertained through the implementation of histological staining and measurement of the diameter of the convoluted seminiferous tubules. Initially, in order to assess the quality of the sample, Pearson correlation analysis was employed to detect significantly lower correlations between calves and adult bovines. This finding was then contrasted with the high correlation coefficients observed between samples within the group (Fig. S1A and Fig. S1B). Unsupervised clustering and UMAP dimensionality reduction identified 16 distinct cell clusters (Fig. S1C). Thereafter, they were classified into four major germ cell types: Spermatogonia, Spermatocytes, Round Spermatid, and Elongated Spermatid, by using well known markers, including *UCHL1*,* SYCP3*,* CSTL1*,* PRM1*,* PRM2*,* TNP1*, and *TNP2* [7, 19–21].

**Fig. 1:**
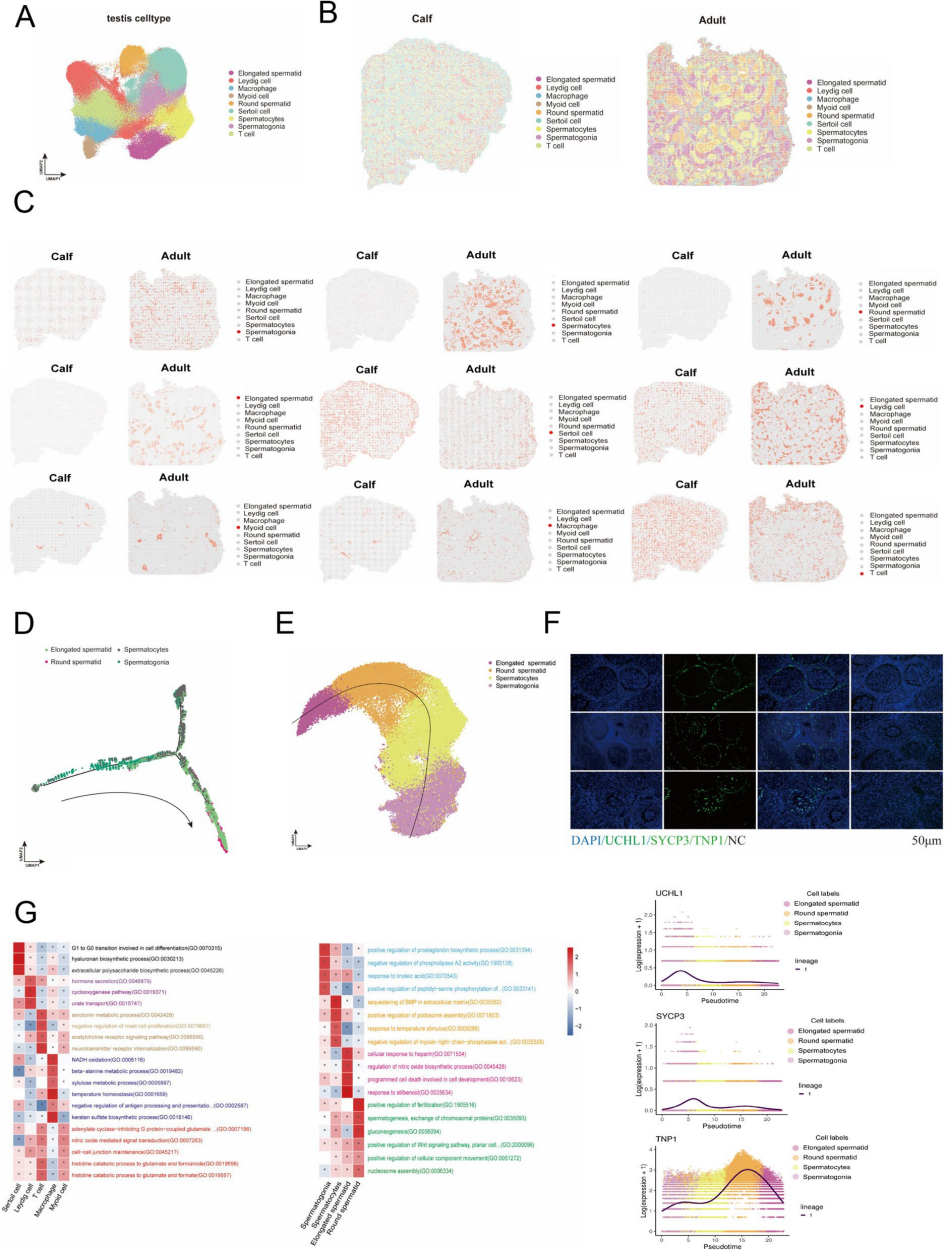
Construction of a spatial transcriptional map of bovine testis tissue. **A** UMAP of bovine testicular tissue, stained differently according to different cell types. Scale bar resolution: 0.4 µm. **B** Spatial slice mapping of testicular cell type distribution included Spermatogonia, Spermatocyte, Round spermatid, Elongated spermatid, Leydig cell, Sertoli cell, Myoid cell, Macrophage, T-cell. Scale bar resolution: 0.4 µm. **C** Mapping of each testicular cell type on spatial sections. Scale bar resolution: 0.4 µm **D** Process analysis of spermatogenesis by Monocle. **E** Simulation of sperm development trajectory by slingshot. **F** Temporal expression and fluorescence localization of UCHL1, SYCP3 and TNP1. Scale bars = 50 μm. **G** The Gene Ontology (GO) of each cell type highlights the significant biological functions of cell types

The somatic cell markers *SOX9*,* AMH*,* CYP11A1*,* ACTA2*, and *MYH11* were found to be integral to the development of five somatic cell types that play a crucial role in the process of bull testicular tissue development [22–25]. These cell types include Sertoli cells, Leydig cells, Macrophages, Myoid cells, and T cells (Fig. 1A and Fig. S1D). Concurrently, we incorporated select cell marker genes for calibration purposes, thereby mitigating the systematic error associated with clustering (Table S1). Spot quantification revealed that only spermatogonia were present in calf testes, and somatic cells such as Sertoli cells and Leydig cells occupied most of the calf testes, which were involved in the construction of the early testicular microenvironment and supported the development of germ cell as well as the process of spermatogenesis. However, the number of somatic and germ cells in the testis of adult bovine is dynamically balanced and the germ cell type are more comprehensive, presenting a complete spermatogenesis process (Fig. S1E). Cell type mapping onto DNB arrays revealed a spatially organized structure within the seminiferous tubules (Fig. 1B and Fig. S1F). From the basal membrane to the luminal surface, the tubules were sequentially populated by spermatogonia, spermatocytes, and spermatids. Furthermore, Sertoli cells formed tight junctions with the germ cells, while the periphery of the tubules were filled with Leydig cells and Myoid cells (Fig. 1C).

To explore the dynamics of spermatogenesis, we used of Monocle 2 to reconstruct the pseudotemoral trajectory of germ cell development in bovines (Fig. 1D). Given the presence of a single germ cell type in calves, we were able to clarify the development initiation site from spermatogonia to elongating spermatid (Fig. S1G) [26]. Monocle trajectory analysis identified the spermatocyte to round spermatid transition as a critical stage in spermatogenesis, marking the completion of meiosis, chromatin reorganization, organelle remodeling, and the associated reduction in DNA content (Fig. 1D). Further consistent developmental trajectories were obtained by slingshot in combination with downscaled coordinates and cell types (Fig. 1E) [27]. In addition, the marker genes of spermatogonia, spermatocytes, spermatids were also mapped on, and their trajectory localization was consistent with developmental trajectories (Fig. S1H). Pseudotime analysis reveals stage-specific expression: *UCHL1* marks spermatogonia, *SYCP3* is upregulated upon meiosis initiation, and *TNP1* is highly expressed in round spermatids. Following immunofluorescence staining, the localization of its expression was elucidated, as well as its use as a marker gene for bovine germ cells, and we did a negative control to avoid non-specific control (Fig. 1F).

Finally, an analysis was conducted to ascertain the predominant biological processes occurring in each cell type. Regarding germ cells, we found that spermatogonia regulate the synthesis of relevant androgens, which may be involved in initiating meiosis and maintaining the microenvironment of the blood-testis barrier by constructing local signaling pools, and that spermatid cells are involved in cell development and apoptotic processes. Round spermatids are engaged in the process of protein replacement (Fig. 1G). Somatic cells are more likely to assist in the development of germ cell and maintenance of the dynamic balance of the testicular microenvironment (Fig. 1G).

In conclusion, we successfully constructed spatial transcriptional profiles of testicular tissues from calves and adult bulls, revealing their germ cell developmental trajectories as well as major cellular types.

### Characterization of bovine testicular germ cell subpopulations

**Fig. 2:**
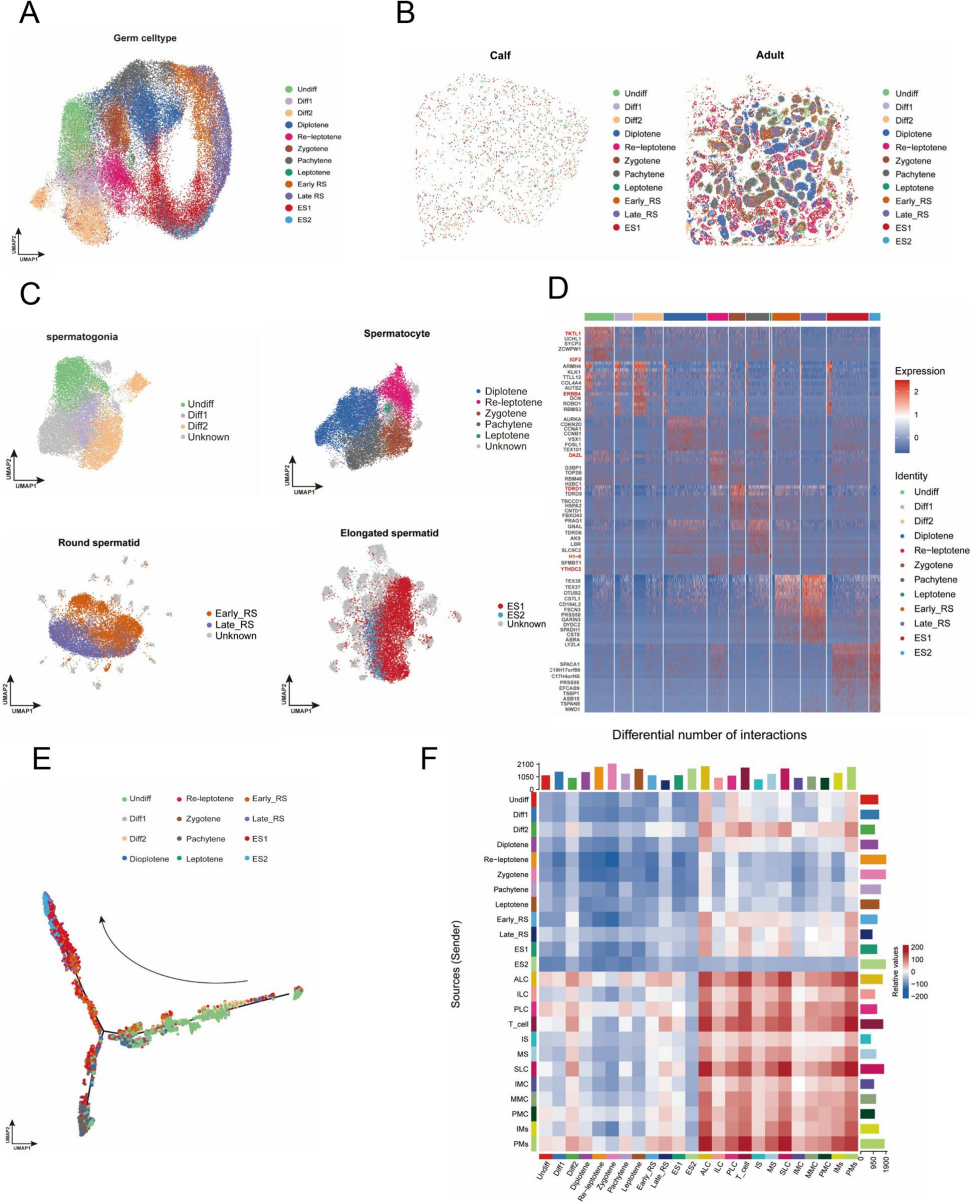
Characterization of bovine testicular germ cell subpopulations. **A** UMAP of bovine germ cell subpopulations, stained differently according to spermatogonia, spermatocyte, round-spermatid, elongated-spermatid. Scale bar resolution: 0.4 µm. **B** Spatial slice mapping of germ cell type. Scale bar resolution: 0.4 µm. **C** UMAP of bovine germ cell subpopulation, stained differently according to different subpopulation types. Scale bar resolution: 0.4 µm. **D** Marker gene heat map of each germ cell subpopulation. **E** pseudo-time sequence trajectory diagram of germ cell subpopulation development by Monocle. **F** Comparison of the number of intercellular communications between calf and adult bovine

To further illuminate the dynamic developmental process of the bovine spermatogenesis lineage, we performed a subsequent dimensionality reduction analysis for germ cell type, yielding a refined UMAP representation of germ cell subtypes (Fig. S2A). Sub-populations were annotated using canonical markers reported in human, mouse and porcine testis atlases. In the initial phase of the study, the spermatogonia were subdivided into three subtypes: Undiff, Diff1, and Diff2. This subdivision was based on the specific expression of *UCHL1* in undifferentiated spermatogonia and the expression of *CD87* and *HSPA8* [24, 28]. With the differentiation of spermatogonia into the first meiotic division, we defined the pre-leptotene and leptotene spermatocyte type by *DAZL, SYCP1*, *SYCP2*. After the spermatocyte passes through the leptotene, genes such as *PIWIL1, H2AZ1*, *CCNA1*, and *CLGN* begin to be expressed, and based on their expression characteristics, we have categorized them into three stages: zygotene, pachytene, and diplotene. Secondary spermatocytes could not be reliably delineated due to their transient nature, lack of a stable transcriptional profile, and exceedingly rapid transition into round spermatids [8, 19, 24, 29]. For spermatids, we classified the developmental stages of these two cell populations based on the expression of the relevant genes; the round spermatid stage defined early round spermatid (early-RS) and late round spermatid (late-RS) by the expression of *CSTL1, TNP1, TNP2, PRM2*. Subsequently, elongated spermatids were categorized into ES1 and ES2 types based on the expression of *TEX29*, *SPAG6*, and *TMEM190*. By mapping the results of the identified cell subpopulations to the UMAP image, we obtained a more comprehensive UMAP image that encompasses all germ cell subpopulation types (Fig. 2A and Fig. S2B) [7, 30, 31].

Mapping the subpopulations back onto tissue sections confirmed their stereotyped spatial order: Undiff spermatogonia adjacent to the basement membrane, meiotic cells in the mid-epithelium, and post-meiotic spermatids approaching the lumen (Fig. 2B). Following this, we extracted each germ cell types and increased the resolution for subpopulation UMAP mapping as a way to understand the transcription level as well as the status among the subpopulations (Fig. 2C). Therefore, we investigated the expression patterns of highly expressed genes within each cell subpopulation for future studies, including the spermatogonia marker gene *TKTL1*, the spermatocytes marker gene *DAZL*, and the genes localized and expressed in spermatids, such as *CST8* and *PRSS55*. Previous studies have reported on these genes in different species (Fig. 2D).

Subsequent to reclustering, we still performed the pseudotime analysis, and the trajectory trend is consistent with the aforementioned findings (Fig. S2C). However, the difference is that this time we corresponded to the refined subpopulation, and ideally the trajectory developmental trend is in line with the developmental characteristics of the germ cell subpopulation. It has been demonstrated that the key node of the pseudotime occurs in the transition from diplotene to early-RS. This finding serves to confirm that the transition from spermatocytes to round spermatids is accompanied by a highly active transcription level (Fig. 2E).

Eventually, to reveal the communication between these subpopulations, especially the differences between calves and adults, a comparative analysis of their cellular interactions was conducted. This analysis revealed that the number and intensity of interactions between testicular somatic cells were augmented in adult bulls compared to calves. This phenomenon relies on a sustained spermatogenesis process and efficient endocrine or even functionality. Furthermore, somatic cellular interactions between calves exhibited a reduced functional demand in comparison to those observed in adult bovines. The adult testis had fewer interactions between somatic-germ cells and among germ cells than calves, but the interactions were stronger. This situation may be related to the involvement of somatic cells in germ cell developmental processes in adults, and the strength of interactions would be higher than in calves because of the greater number of biological processes that adults are required to participate in (Fig. 2F and Fig. S2D).

Thus, we have identified subpopulations of bovine germ cells, explored their potential developmental trajectories, screened for subpopulation marker genes, and investigated interactions between calf and adult subpopulations.

### Heterogeneity of developmental processes in bovine spermatogonia

**Fig. 3:**
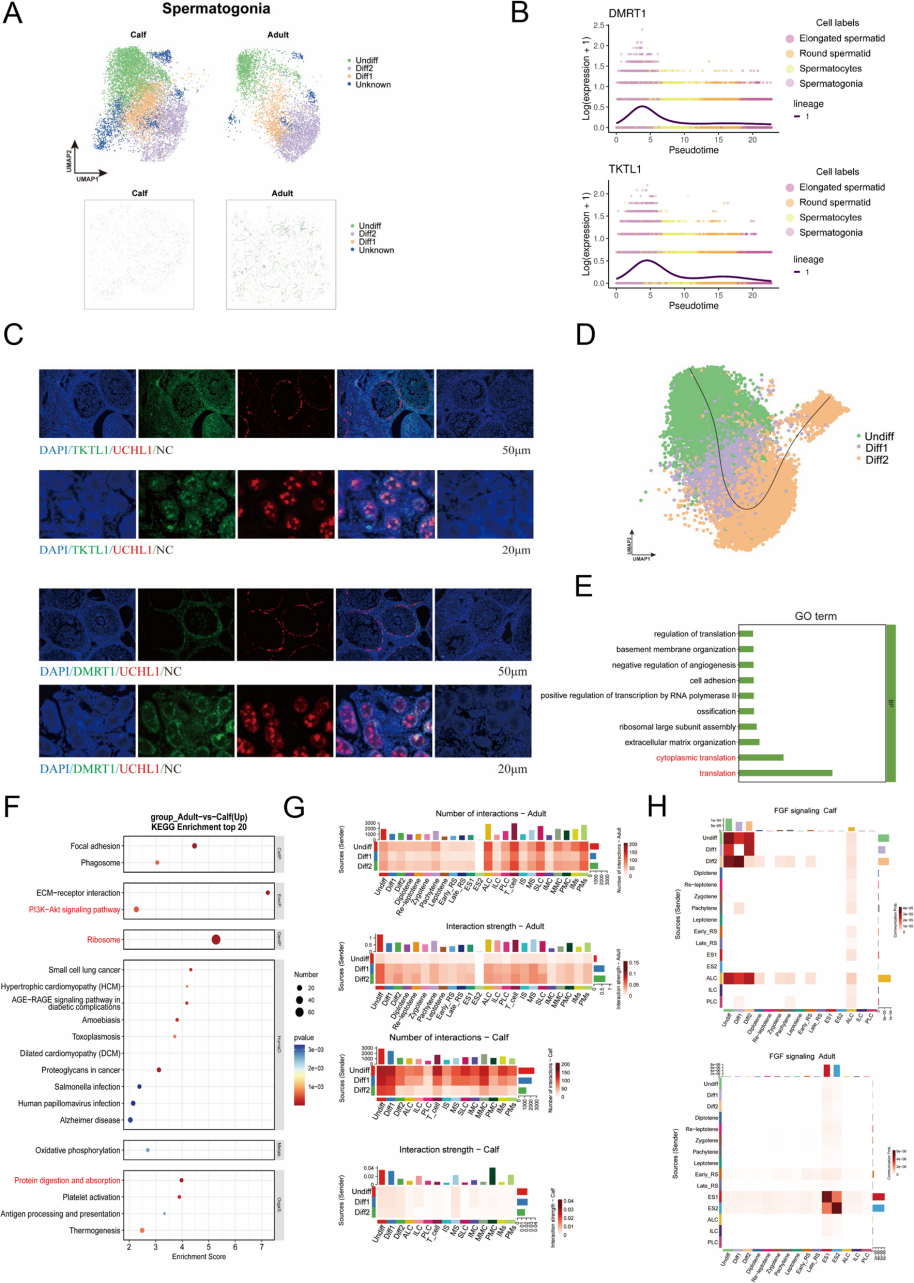
Heterogeneity of developmental processes in bovine spermatogonia. **A** The spatial distribution of spermatogonia subpopulations in calf and adult bovine. Scale bar resolution: 0.4 µm. **B** Timing expression of DMRT1 and TKTL1. **C** DMRT1 with UCHL1 and TKTL1 with UCHL1 in bovine testis. Scale bars = 50 μm. **D** Time sequence development trajectory of spermatogonia subpopulation. **E** Compared with adult bovine, the up-regulation of GO biological process of differential genes in spermatogonia of calves. **F** Compared with adult bovine, the up-regulation of KEGG pathways of differential genes in spermatogonia of calves. **G** The heatmap of number and strength of interaction between bovine spermatogonia and other cells in two stages. **H** Different intensity patterns of FGF signaling pathway in calves and adult bovine

Spermatogonia, the initiating cells of spermatogenesis, play a pivotal role in bovine testicular development. To investigate the functional differences of spermatogonia before and after bovine development, the spermatogonia population of calves and adults were analyzed separately. Initially, a higher percentage of Undiff cell population was observed in calves compared to adult bovine. Conversely, adults were dominated by the committed Diff2 subset, indicative of an active differentiation program, the spermatogonia in this period are ready to differentiate into spermatocytes (Fig. S3A). The same results were shown by UMAP image and mapping to sections, and the distribution of spermatogonia on sections was not as regular in calves as in adult bovine due to the less well-developed development of the seminiferous tubules in calves compared to adult bovine (Fig. 3A).

Thereafter, we analyzed the temporal expression of some spermatogonia marker genes and found that the expression of *ERBB4, DMRT1, TKTL1* progressively declined in spermatogonia and was highly expressed in the Undiff cell population. The study concluded with the determination that these genes are specifically expressed in spermatogonia populations (Fig. 3B and Fig. S3B). To verify this, we performed double immunofluorescence staining of DMRT1/UCHL1 and TKTL1/UCHL1, which showed that both DMRT1^+^ cells and TKTL1^+^ cells overlapped with the undifferentiated spermatogonia cell marker UCHL1. Consistent staining results were observed in calves and adult bovine, except that it was evident that DMRT1 was localized in the nucleus, whereas TKTL1 was localized in the cytoplasm (Fig. 3C). A trajectory developmental analysis of spermatogonia subpopulations is conducted. The developmental processes under analysis range from Undiff to Diff2, and the analysis is based on spermatogonia temporal developmental changes (Fig. 3D). In addition to marker genes, we identified co-expression regulon of transcription factors with potential target genes by SCENIC and correlated them specifically for spermatogonia cell type [32]. KDM5B, a potential transcription factor for spermatogonia cells, may play a role in DNA repair and genome stability (Fig. S3C) [33].

We then analyzed the differential genes (DEGs) between adult and calf spermatogonia, with 377 up-regulated and 727 down-regulated genes, followed by GO and KEGG enrichment analyses (Fig. S3D and Fig. S3E). It was found that compared to calf spermatogonia, the up-regulated expression in adult bovine was mainly focused on the process of protein synthesis-translation. In combination with the cellular components, it was revealed that the spermatogonia of adult bovine were in a more mature state to meet their specific protein requirements (Fig. 3E). Beyond ribosomal pathways, adult bovine spermatogonia showed upregulation in energy metabolism as well as proliferation and growth pathways, clarifying the functional distinctions between the two developmental stages (Fig. 3F).

A notable finding emerged from the analysis of spermatogonia interactions, which revealed that spermatogonia interactions were more numerous in calves than in adults. This elevated interaction rate was attributable not only to the predominance of undifferentiated spermatogonia in calves but also to the fact that the calf testis is in the initiation phase of puberty. During this phase, the testis must establish developmental pathways not only to itself but also to the somatic cells in an extensive manner (Fig. 3G). For the strength of interactions, due to the refinement of the Blood-Testis Barrier (BTB) in adult bovines, the efficiency of signal transmission increases, enhancing ligand-receptor binding and increasing interaction strength (Fig. 3G). Subsequently, we selected the FGF signaling pathway, a classical signaling pathway in spermatogonia, to study, and obtained the result that FGF signaling was stronger in calf spermatogonia, corresponding to its efficient self-renewal. Instead, FGF signaling was stronger in spermatid of adult bovines, which has been shown to be associated with morphological changes and function of spermatid (Fig. 3H) [34]. In calves, the proportion of FGF8/FGFR1 signal is high, which is helpful for the differentiation of stem cells, and the proportion of FGF1/FGFR2 signal is high in adults (Fig. S3F).

In conclusion, we screened and validated marker genes for bovine testicular spermatogonia and screened for spermatogonia population-specific transcription factors. A comparison of functional differences in spermatogonia between calves and adult bovines was conducted, revealing the heterogeneity of the differentiation process of bovine spermatogonia.

### Characterization of spatial transcription in bovine spermatocyte revealed

**Fig. 4:**
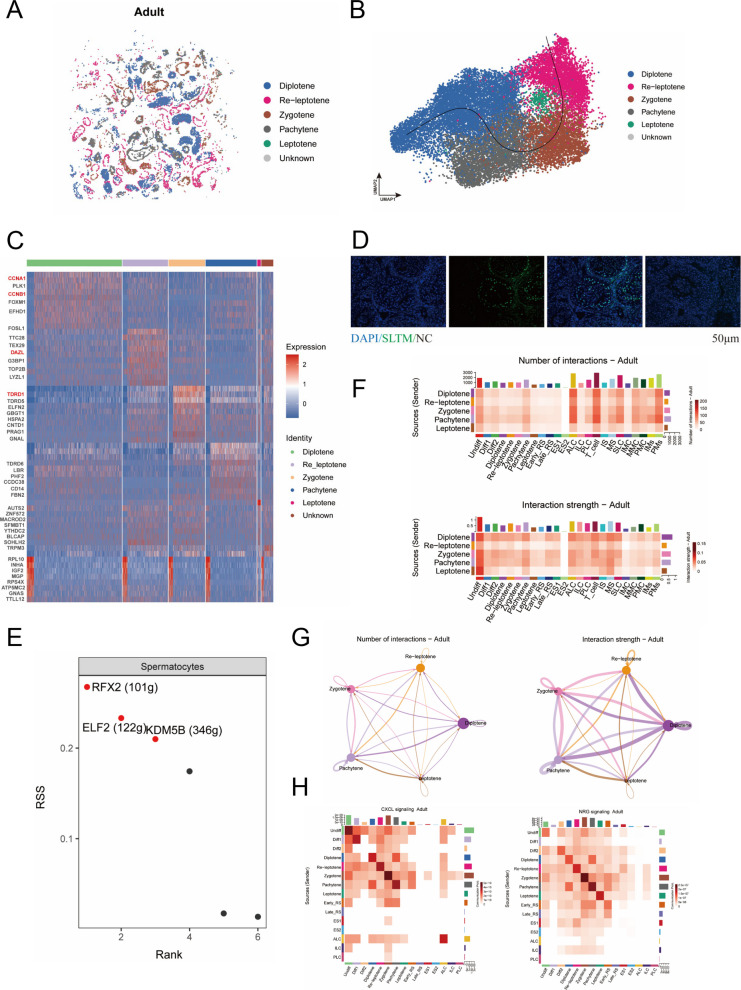
Characterization of spatial transcription in bovine spermatocyte revealed. **A** The spatial distribution of spermatocytes subpopulations in adult bovine. Scale bar resolution: 0.4 µm. **B** Time sequence development trajectory of spermatocyte subpopulation. **C** Marker gene heatmap of spermatocyte subpopulation. **D** Localization of SLTM in bovine testis. Scale bars = 50 μm. **E** Regulon specificity score (RSS) of spermatocytes. **F** The heatmap of number and strength of interaction between bovine spermatocytes and other cells. **G** The number and strength network diagram of interaction between spermatocytes. **H** The interaction pattern of CXCL and NRG signaling pathways

In light of the observed absence of development in the calf spermatocytes and spermatid, subsequent studies were directed towards the testes of adult bovines. Firstly, the various subpopulations of spermatocytes were mapped onto tissue sections, and it was ascertained that the majority of spermatocytes were in the pachytene and diplotene phases (Fig. 4A). In a similar manner, these five spermatocyte subpopulations were subjected to trajectory developmental analysis, undergoing a progression from leptotene through zygotene and pachytene, ultimately culminating in diplotene development (Fig. 4B). The expression of marker genes at each spermatocyte stage is crucial, as these genes serve as key molecular tags for tracking meiotic progression. For this reason, we characterized the expression of marker genes in spermatocytes at various stages (Fig. 4C).

Concurrently, we validated one of the identified spermatocyte markers and observed that SLTM^+^ cells were localized to spermatocytes. Our results confirm its utility as a marker gene for bovine spermatocytes (Fig. 4D and Fig. S4A). The intricacy of the meiotic process, which entails the coordinated action of numerous regulators, necessitated the formulation, necessitated the development of a comprehensive prediction model for the transcription factors of the spermatocyte type. The model demonstrated a significant correlation between RFX2 transcriptional regulation and the spermatocyte population. Notably, RFX2 has been identified as a pivotal transcriptional regulator of the meiotic process in spermatogenesis in mice (Fig. 4E) [35, 36]. Then we predicted and screened the target genes of RFX2. A total of 60 target genes were screened for subsequent studies using a combined analysis of multiple databases (Fig. S4B).

Furthermore, an investigation was conducted into the interactions of spermatocytes within the adult bovine testis. The results of this investigation indicated that the number of interactions between spermatocytes and various types of cells is not particularly substantial. However, for the strength of the interactions there is a much higher level of zygotene and pachytene. This phenomenon underscores the critical role of effective communication in coordinating complex chromosomal events (Fig. 4F). The separation of spermatocytes revealed that the strength of spermatocyte interactions increased during all stages of late meiosis I, especially self-communication among diplotene spermatocytes (Fig. 4G). In order to deeply explore the above phenomenon of high interaction strength in spermatocyte, we have resolved the key signaling pathways of spermatocyte meiosis. The CXCL signaling pathway was found not only to have high-intensity communication in spermatogonia [37], but also revealed its equally high reciprocal strength in spermatocytes, especially in the zygotene and pachytene (Fig. 4H and Fig. S4C). Our findings indicate a high intensity expression of NRG signaling, which binds to ERBB4 to induce meiosis in spermatocytes (Fig. 4H and Fig. S4D) [37].

In short, we verified the rationality of spermatocyte subpopulation subgroups by cell developmental trajectories, and identified marker genes for each stage of spermatocyte meiosis, screened a spermatocyte marker SLTM and verified it by staining, and revealed transcriptional features of bovine spermatocytes in meiosis.

### Dynamic transcription expression patterns during bovine sperm deformation

**Fig. 5:**
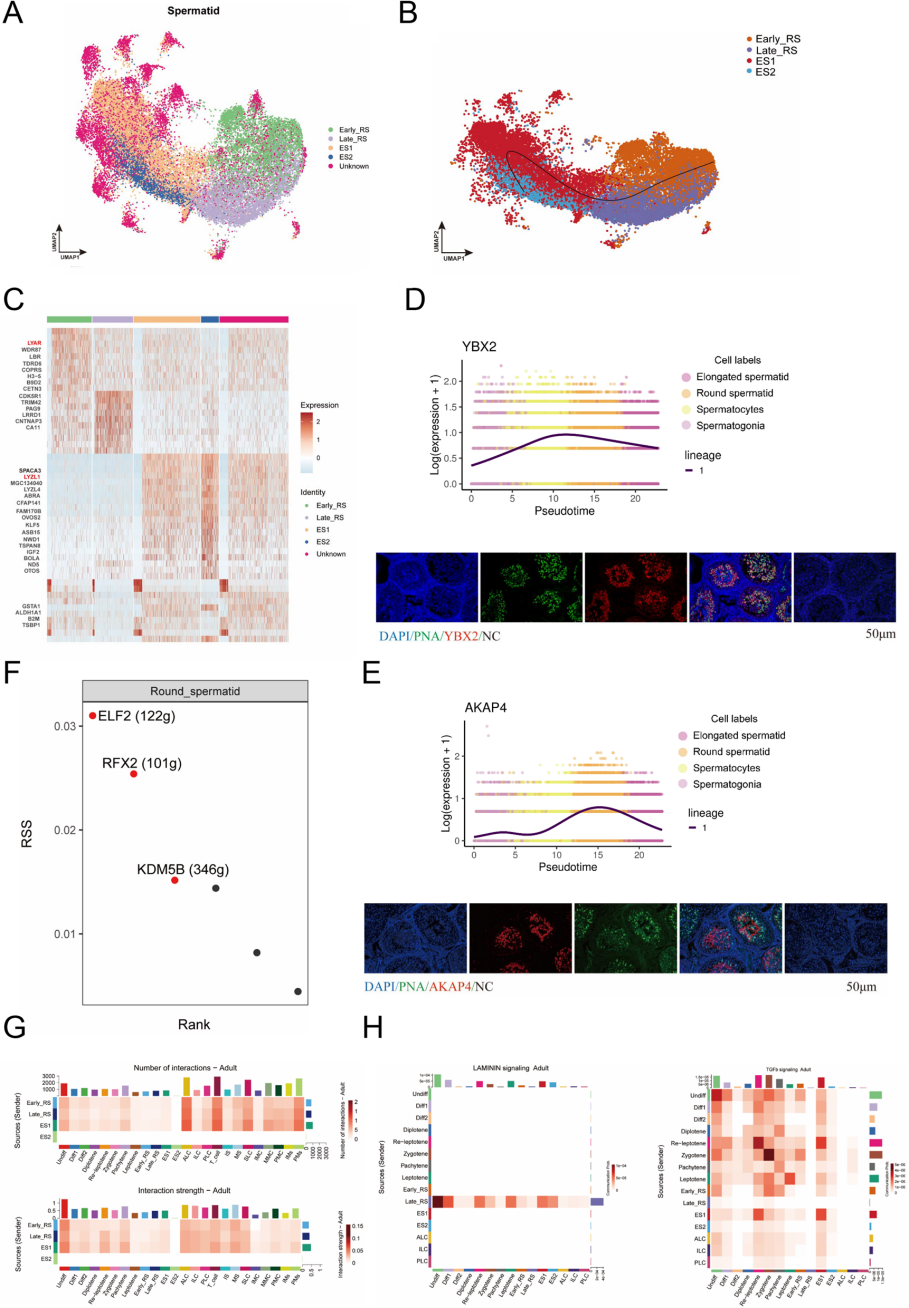
Dynamic transcription expression patterns during bovine sperm deformation. **A** UMAP of bovine spermatid subpopulation, stained differently according to different subpopulation types. Scale bar resolution: 0.4 µm. **B** Time sequence development trajectory of spermatid subpopulation. **C** Marker gene heatmap of spermatid subpopulation. **D** The temporal expression pattern of YBX2, and YBX2 with PNA in bovine testis. Scale bars = 50 μm. **E** The temporal expression pattern of AKAP4, and AKAP4 with PNA in bovine testis. Scale bars = 50 μm. **F** Regulon specificity score (RSS) of round spermatids. **G** The heatmap of number and strength of interaction between bovine spermatids and other cells. **H** The interaction pattern of LAMININ and TGFb signaling pathways

The transformation of round spermatids into mature spermatozoa involves extensive morphological remodeling. This stage of development is regulated by specific genes to ensure proper sperm formation and functioning. In order to facilitate a more continuous study of the sperm deformation stages, round and elongated spermatids were combined and re-clustered to generate a unified UMAP representation containing only sperm cells (Fig. 5A). The recently developed cell lineage map was subject to analysis for sperm cell differentiation trajectories from Early-RS to Late-RS, which from a flagellum and subsequently develop into ES1 to ES2. In the analysis, the interference of the unknown cell type was excluded from the results (Fig. 5B).

Next, the marker genes were examined at four stages, revealing that *LYAR* is present in spermatocytes and early spermatid cells but absent from spermatids, which can be used as an Early-RS marker [38]. LYZL4, a sperm-associated protein found in mice, plays a role in fertilization and is present in more mature spermatids. Two of the genes screened above can be used for subsequent studies (Fig. 5C) [39]. The YBX2 marker was selected to provide a more comprehensive identification of spermatid cells. While initial transcriptomic data detected YBX2 expression in earlier stages (spermatogonia and spermatocytes), immunofluorescence staining definitively localized the YBX2 protein exclusively to spermatids. This finding suggests the presence of post-transcriptional regulation, whereby transcription occurs earlier but translation into protein is only initiated during the spermatid stage. We used the acrosomal marker PNA to co-stain with YBX2 and observed that PNA^+^ cells localized in the same cell type as YBX2^+^ cells just YBX2 was localized in the cytoplasm, which proved that *YBX2* could be used as a spermatid cell marker gene (Fig. 5D and Fig. S5A) [40]. AKAP4 is one of the more abundant proteins in spermatids, and its presence is essential for flagellar development. Its expression began to increase during the round spermatid period, as evidenced by psedotime. Furthermore, the results of the fluorescence analysis demonstrated its localization in the sperm flagellum, which can be used as a marker gene for late spermatids (Fig. 5E and Fig. S5A) [41]. Consequently, an exhaustive search for transcriptional regulators of spermatids was conducted, leading to the identification of the transcription factor CREM for round spermatid. This analysis also yielded the prediction of three downstream target genes (Fig. 5F and Fig. S5B) [42]. Given that elongated spermatid are more proximate to mature sperm, their transcriptional regulation is reduced, therefore, we did not predict them.

Round spermatids are in the metamorphosis phase of sperm, establish close communication with T cells and Leydig cells to harmonize development and immune homeostasis. Leydig cells have been demonstrated to drive the development of round spermatid through testosterone, while round spermatid, in turn, regulate Leydig cell function through the use of feedback signals (Fig. 5G) [43]. It is noteworthy that ES2 interactions were not observed in elongated spermatid, a finding that may be associated with the elevated concentration of sperm chromatin and the termination of transcription, resulting in the incapacity to synthesize novel signaling molecules (Fig. S5C). A comprehensive analysis of specific signaling has led to the identification of a high-intensity interaction of the LAMININ signal during the round spermatid stage. This finding suggests that the proteins secreted by round spermatids regulate spermatogenesis, by modulating processes such as trans-epithelial transport of sperm cells and BTB dynamics [44]. TGF signaling has been demonstrated to be highly effective in promoting sperm formation; however, it should be noted that this signaling pathway is also strongly active in other cell populations. The report has demonstrated that this signaling pathway is an indispensable component of testis formation and participates in various biological processes within the testis (Fig. 5H and Fig. S5D) [45, 46].

The results of our study demonstrate significant transcriptional differences during the transition from round spermatid to elongated spermatid. Furthermore, we have identified and validated two marker genes that are specifically expressed in sperm cells. The present study elucidates the function of round spermatid secretory proteins and gradual cessation of transcription in late elongated sperm.

### Spatial transcriptional characteristics of bovine testicular somatic cell subpopulations

**Fig. 6:**
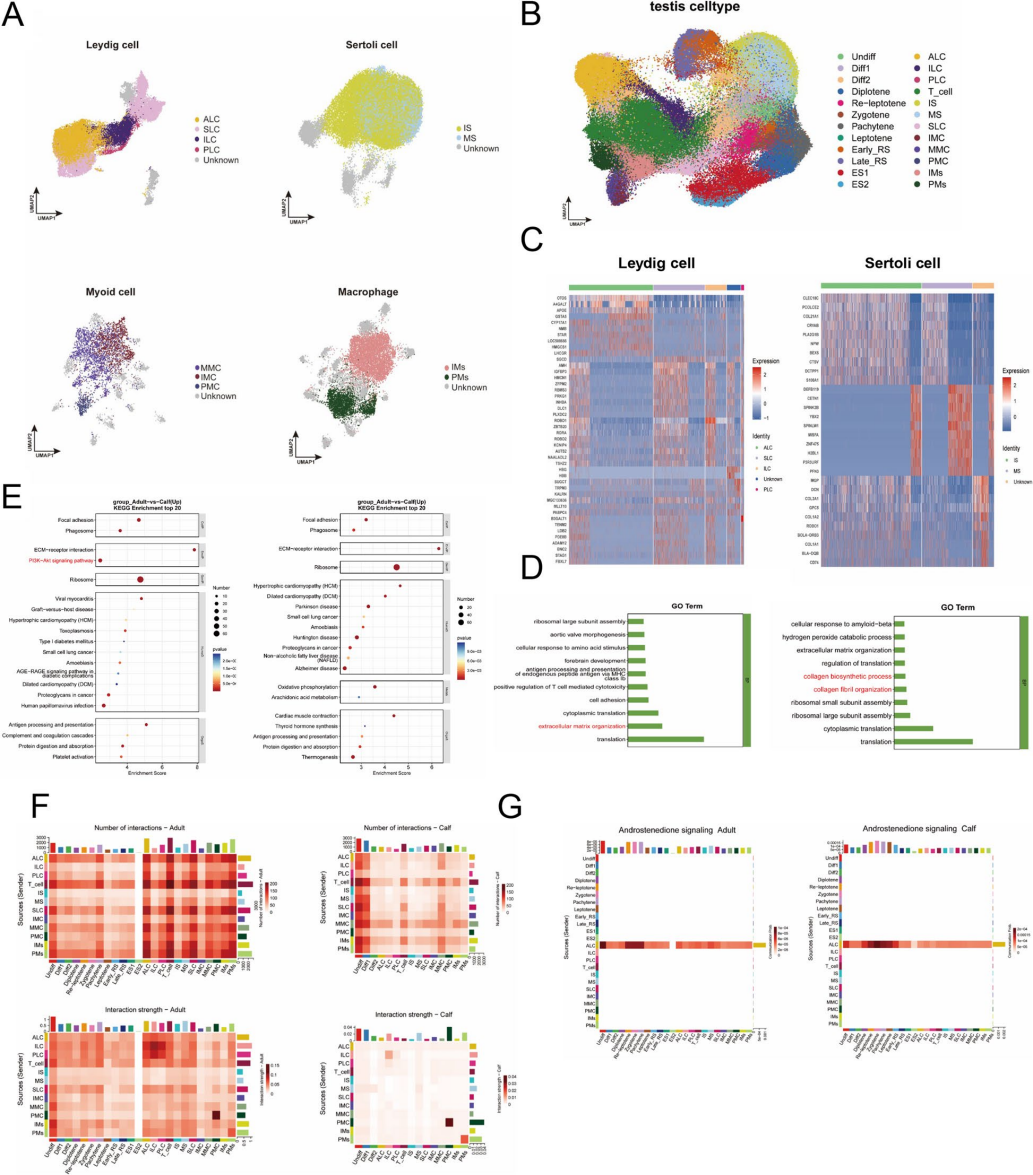
Spatial transcriptional characteristics of bovine testicular somatic cell subpopulations. **A** UMAP of bovine somatic cell subpopulation, stained differently according to different subpopulation types. Scale bar resolution: 0.4 µm. **B** UMAP of bovine testicular cell subpopulation, stained differently according to different subpopulation types. Scale bar resolution: 0.4 µm. **C** Marker gene heatmap of Leydig cell and Sertoli cell subpopulation. **D** Compared with adult bovine, the up-regulation of GO biological process of differential genes in Leydig cell (left) and Sertoli cell (right) of calves. **E** Compared with adult bovine, the up-regulation of KEGG pathways of differential genes in Leydig cell (left) and Sertoli cell (right) of calves. **F** The heatmap of number and strength of interaction between bovine somatic cell and other cells in two stages. **G** The interaction pattern of Androstenedione signaling pathway in two ages

In addition to germ cells, somatic cells play a critical role in the development of bovine testes. To investigate how these somatic cells evolve and develop and assist in the process of spermatogenesis, we divided the important somatic cell populations into subgroups. Progenitor myoid cells (PMC), immature myoid cells (IMC), and mature myoid cells (MMC) were distinguished based on the expression of MYH11, TAGLN, and ACTG2. The low expression of IMC was identified as the active symbol distinguishing them from MMC [24]. The elevated expression of CYP11A1 and CYP17A1 was instrumental in the successful differentiation of adult Leydig cells (ALC). Conversely, low CYP11A1 and CYP17A1 expression and high ROBO1/2 expression delineated immature Leydig cells (ILC). Moreover, high expression of PDGFRA indicated their elevated proliferative capacity, these cell populations were identified as progenitor Leydig cells (PLC) [47] Finally, we categorized Sertoli cells as immature (IS) or mature (MS) based on AMH expression. Additionally, macrophages were classified by the function of relevant genes into two subsets: interstitial macrophages, which are involved in the immune response and support testosterone secretion, and peritubular macrophages, which are associated with spermatogenesis (Fig. 6A and Fig. S6A) [48].

Having defined these somatic cell subpopulations, we next visualized their spatial relationships and developmental shifts by mapping them onto the UMAP projection (Fig. 6B). In calves, both ALC and IS exhibited broad dispersion, indicating that the primary role of Leydig cells is hormone production-testosterone. This functional priority correlates with a higher abundance of mature Leydig cells. Conversely, Sertoli cell maturation depends on BTB formation, through which they secrete androgen-binding protein (ABP) to maintain local levels of Leydig-derived hormones (Fig. S6B). At the same time, we screened for marker genes of Leydig cells and Sertoli cells, with no significant differences observed between the IS and MS subtypes of Sertoli cells (Fig. 6C).

Additionally, we identified the differentially expressed genes in somatic cells between calves and adult, and subsequently performed GO and KEGG pathway analyses. From a biological process, the Leydig cells of adult bovines primarily engage in the synthesis and organization of the extracellular matrix, a process that facilitates the maintenance of Leydig call functionality [49]. The present study demonstrated that Sertoli cells increased collagen synthesis and related regulatory processes. Research has demonstrated that the collagen chains produced by Sertoli cells regulate the functions of different stages of the spermatogenic epithelium cycle (Fig. 6D) [50]. Concurrently, KEGG pathway analysis revealed differential activation of specific pathways, showing that adult bovine Leydig cells, compared to their counterparts in calves, exhibited enhanced activity in protein synthesis, key growth-promoting signals, and interactions with the extracellular matrix. These findings suggest that adult bovine Leydig cells and Sertoli cells are more mature than those found in calf cells. Adult bovine somatic cells exhibit vigorous metabolism and robust barrier function, reflecting the stable spermatogenic environment maintained within adult bovine testes (Fig. 6E).

Through interaction analysis, it was determined that the number of interactions between somatic cells in calves is lower than that observed in adult bovine. Additionally, lower interaction strength in calves further indicates that the somatic cell microenvironment in adult bovine testes is more mature (Fig. 6F). We identified a specific signaling pathway for ALC, the Androstenedione signaling pathway, which serves as an intermediate for testosterone and can only be converted into testosterone by 17β-HSD3, an enzyme expressed by ALC. This signaling pathway plays a critical role in regulating the dynamic balance of testosterone, maintaining the testicular microenvironment, and enhancing sperm production in both calves and adult bovine (Fig. 6G and Fig. S6C) [51].

## Discussion

Spermatogenesis is a highly orchestrated process governed by intrinsic and extrinsic cues. The dynamic transcriptional processes within the seminiferous tubules warrant further investigation. Spatial transcriptional maps of the testis at molecular resolution have been characterized in mice and humans; however, information regarding large mammals remains partially unexplored [48, 52]. In this study, we employed Stereo-seq to construct spatial transcriptomic atlases of testicular tissues from both calves and adult bovine. Given the limitations of the samples, we define that pre-sexually mature calves lack a complete spermatogenesis process, while sexually mature adult bovine can stably produce sperm cells. Despite all this, our findings elucidated gene expression changes and alterations in biological processes during bovine spermatogenesis. We further identified subtypes of germ and somatic cells, refining the continuum of bovine spermatogenesis. The spatial dynamics of gene expression and cellular composition within seminiferous tubules were systematically evaluated.

### Definition of germ cell subpopulations

The identification of germ cell subpopulations has been completed based on existing marker genes. In comparison with the finding of previous studies on single-cell sequencing, our results indicate that subpopulations are subdivided into a more continuous process displayed on spatial slices.

Interestingly, spermatogenesis is a spatially continuous process, so the distinction between subpopulations is made from a developmental perspective. This mirrors the classical distinction between undifferentiated and differentiating spermatogonia, the meiotic process of spermatocytes, and spermatid before and after morphogenesis. However, the definition of these subpopulations remains limited. To illustrate this point, we may consider spermatogonia, which are traditionally classified into seven distinct types: As, Apr, Aal, and A1–A4 ,with the former maintaining spermatogonia stem cell function and the latter tending toward differentiation. The precise definition of these subpopulations is challenging due to the absence of marker genes and resolution issues with current analytical techniques [53, 54]. Therefore, this study offers empirical evidence that substantiates the delineation of male germ cell subpopulations. This development paves the way for further investigation into their functional roles and facilitates the development of more precise subpopulation characterization methodologies in the future.

### Development of germ cell markers

In our study, the validation of five germ cell markers was conducted. To this end, we employed immunofluorescence to investigate the localization of DMRT1, TKTL1, SLTM, YBX2, and AKAP4 in bovine testes. Our findings indicated that DMRT1 and TKTL1 can serve as spermatogonia cell markers, and SLTM can function as a spermatocyte marker, and YBX2 and AKAP4 can be utilized as spermatid cell markers. A particularly noteworthy aspect is the localization of spermatogonia cell markers in undifferentiated spermatogonia cells.

*DMRT1*, a Z chromosome-linked gene in birds, has been demonstrated to contribute to the differentiation of the male sex in avian species. In contrast, in mammals, DMRT1 functions as a transcription regulatory factor necessary for maintaining spermatogonia stem cells [55, 56]. In subsequent research, elucidating the regulatory mechanism of DMRT1 on spermatogonia cells will be a significant undertaking.

TKTL1 is a transketolase enzyme involved in sugar metabolism and serves as a tumor marker [57]. In the current study, single-cell sequencing was performed on goat testicles, and *TKTL1* was identified as a potential marker gene for spermatogonia; however, this has not yet been proven. Our results support this finding and suggest that TKTL1 could be considered a spermatogonia marker in ruminants [58].

In summary, our results will aid in developing sorting markers and provide theoretical support for subsequently sorting germ cells.

### Regulation of male germ cell by transcription factors

In this study, we sought to predict transcription regulatory factors for various germ cells through the analysis of co-expression modules between genes. KDM5B (JARID1B), predicted in spermatogonia cells, is a classic transcription regulatory factor, essentially a histone demethylase. In mice, KDM5B has been found to interact with the androgen receptor as a transcription factor and regulate its transcriptional activity [59]. As indicated by the present findings, RFX2 functions as a transcriptional regulator in spermatogenesis, primarily regulating the expression of *H1t* and related genes during meiosis [35, 36, 60]. The cAMP response element modulator (CREM) is imperative for the development of spermatid cells into sperm, thereby underscoring its crucial role in transcriptional regulation of sperm cells [61].

However, due to the limitations of the current in vitro culture system for bovine germ cells, which severely hinder the study of the functional mechanisms of transcription factors, the focus of our research is twofold: improving the germ cell sorting system and establishing an in vitro culture model. In subsequent studies, we will investigate the mechanisms by which transcription factors regulate bovine germ cells using the enhanced culture system.

### The function of the FGF signaling pathway at different stages of spermatogenesis

Fibroblast growth factor (FGF) is widely regarded as a hormone factor. Research has demonstrated that FGF plays a pivotal role in regulating testicular development, with multiple members of the FGF family implicated in this process [62].

Interestingly, diverse FGF signaling factors regulate distinct biological processes at various stages. In mouse spermatogonia stem cells, FGF2 induces the phosphorylation of AKT and MAPK1 and upregulates the expression of genes such as *Etv5* and *Bcl6*, and thereby maintains the self-renewal of spermatogonia stem cells [63]. Notably, not only does FGF maintain the continuous production of spermatogonia cells, but FGF9/FGF7 can activate downstream signals SOS1 and FRS2 to promote spermatogonia cell differentiation [64]. However, compared to the childhood stage, FGFR1/FGFR2 exhibits elevated levels of expression in mature sperm, thereby regulating sperm motility and function [65].

In consideration of the results obtained, it can be concluded that the FGF signaling pathway plays a predominant role in the establishment of the spermatogonia pool within the context of calf spermatogonia. In adult bovines, FGF signaling is prominently active in elongated spermatids, where it may regulate flagellar function, suggesting that FGF plays a primary role in regulating sperm motility and vitality at this stage. Consequently, we observed divergent interactive patterns of the FGF signaling pathway at varying stages of testicular development.

## Conclusions

Using Stereo-seq technology, we constructed a spatial transcriptomic atlas of testicular cells and identified four germ cell and five somatic cell subtypes. Our findings enable a precise characterization of testicular cell populations and validate five novel germ cell markers in cattle, thereby facilitating a deeper understanding of bovine spermatogenesis. Collectively, this dataset delineates the transcriptional dynamics during bovine spermatogenesis and provides a theoretical foundation for elucidating the mechanisms underlying spermatogenesis in large male mammals.

## Supplementary Information

Additional file 1. Spatial transcriptomics reveals dynamic transcriptional processes in bovine testicular cells. Fig. S1. Construction of a spatial transcriptional map of bovine testis tissue. Fig. S2. Construction of a spatial transcriptional map of bovine testis tissue. Fig. S3. Heterogeneity of developmental processes in bovine spermatogonia. Fig. S4. Characterization of spatial transcription in bovine spermatocyte revealed. Fig. S5. Dynamic transcription expression patterns during bovine sperm deformation. Fig. S6. Spatial transcriptional characteristics of bovine testicular somatic cell subpopulations.

Additional file 2. Marker genes.

## Data Availability

All data that support the findings of this study are openly available in, reference numbers GSE307496 of NCBI.

## Abbreviations

ABP: Androgen-binding protein
ALC: Adult Leydig cells
BTB: Blood-testis barrier
CID: Coordinated identifier
DNB: DNA nanoball
GO: Gene Ontology
ILC: Immature Leydig cells
IMC: Immature myoid cells
IS: Immature Sertoli cells
KEGG: Kyoto Encyclopedia of Genes and Genomes
MID: Molecular identifier
MMC: Mature myoid cells
MS: Mature Sertoli cells
OCT: Optimal cutting temperature
PBS: Phosphate-buffered saline
PCA: Principal component analysis
PLC: Progenitor Leydig cells
PMC: Progenitor myoid cells
RA: Retinoic acid
RNA-seq: RNA sequencing
UMAP: Uniform manifold approximation and projection
