# Supplementary material for: Dissecting the development of bovine testicular tissue using spatial transcriptomics
Source: J Anim Sci Biotechnol. 2026 Feb 5;17:21. doi: 10.1186/s40104-025-01340-4 (PMC12874701; doi:10.1186/s40104-025-01340-4)
Supplement: Supplementary file 1 — Additional file 1. Spatial transcriptomics reveals dynamic transcriptional processes in bovine testicular cells. Fig. S1. Construction of a spatial transcriptional map of bovine testis tissue. Fig. S2. Construction of a spatial transcriptional map of bovine testis tissue. Fig. S3. Heterogeneity of developmental processes in bovine spermatogonia. Fig. S4. Characterization of spatial transcription in bovine spermatocyte revealed. Fig. S5. Dynamic transcription expression patterns during bovine sperm deformation. Fig. S6. Spatial transcriptional characteristics of bovine testicular somatic cell subpopulations. [file 40104_2025_1340_MOESM1_ESM.pdf]

Figure S1

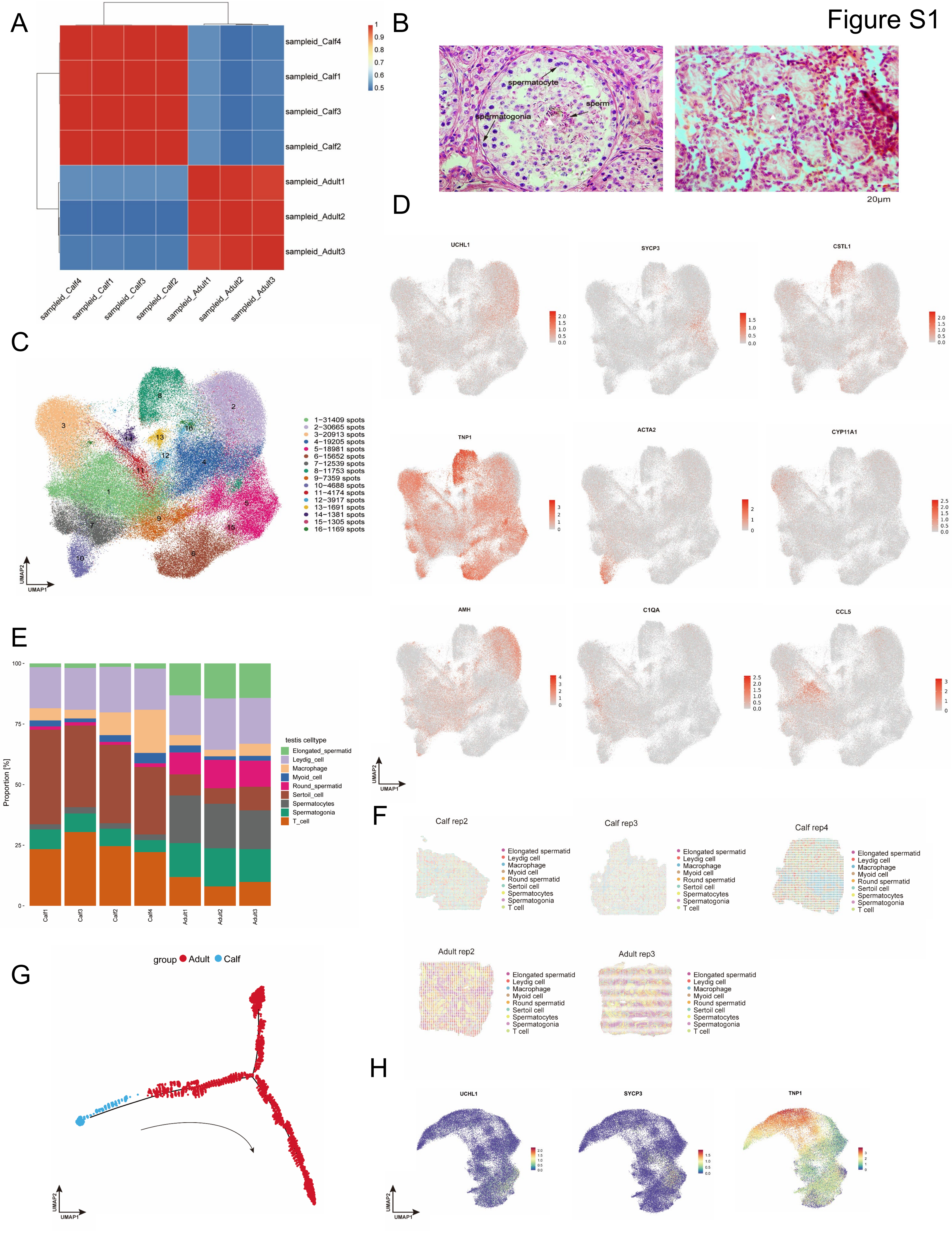
A
sampleid_Calf4
sampleid_Calf1
sampleid_Calf3
sampleid_Calf2
sampleid_Adult1
sampleid_Adult2
sampleid_Adult3
B
spermatocyte
sperm
spermatogonia
20μm
C
1−31409 spots
2−30665 spots
3−20913 spots
4−19205 spots
5−18981 spots
6−15652 spots
7−12539 spots
8−11753 spots
9−7359 spots
10−4688 spots
11−4174 spots
12−3917 spots
13−1691 spots
14−1381 spots
15−1305 spots
16−1169 spots
UMAP1
UMAP2
D
UCHL1
SYCP3
CSTL1
TNP1
ACTA2
CYP11A1
AMH
C1QA
CCL5
E
Proportion [%]
testis celltype
Elongated_spermatid
Leydig_cell
Macrophage
Myoid_cell
Round_spermatid
Sertoil_cell
Spermatocytes
Spermatogonia
T_cell
Calf1
Calf3
Calf2
Calf4
Adult1
Adult2
Adult3
F
Calf rep2
Calf rep3
Calf rep4
Adult rep2
Adult rep3
Elongated spermatid
Leydig cell
Macrophage
Myoid cell
Round spermatid
Sertoil cell
Spermatocytes
Spermatogonia
T cell
G
group Adult Calf
H
UCHL1
SYCP3
TNP1

Fig S1 Construction of a spatial transcriptional map of bovine testis tissue

A Pearson Correlation Index between Sample Repetitions. **B** H&E-stained testicular tissue from calves and adults, including assessment of seminiferous tubule development and germ cell types. bars=20μm. **C** Spatial localization of marker genes on the UMAP plot. **D** UMAP of bovine testicular tissue, stained differently according to different cell types, total of 16 cell populations. Scale bar resolution: 0.4 μm. **E** The proportion of each testicular cell group among the samples. **F** Spatial slice mapping of testicular cell type distribution (biological repetition). **G** The pseudo-time development process of the sample group by Monocle. **H** Expression patterns of UCHL1, SYCP3, TNP1 in UMAP plots.

Figure S2

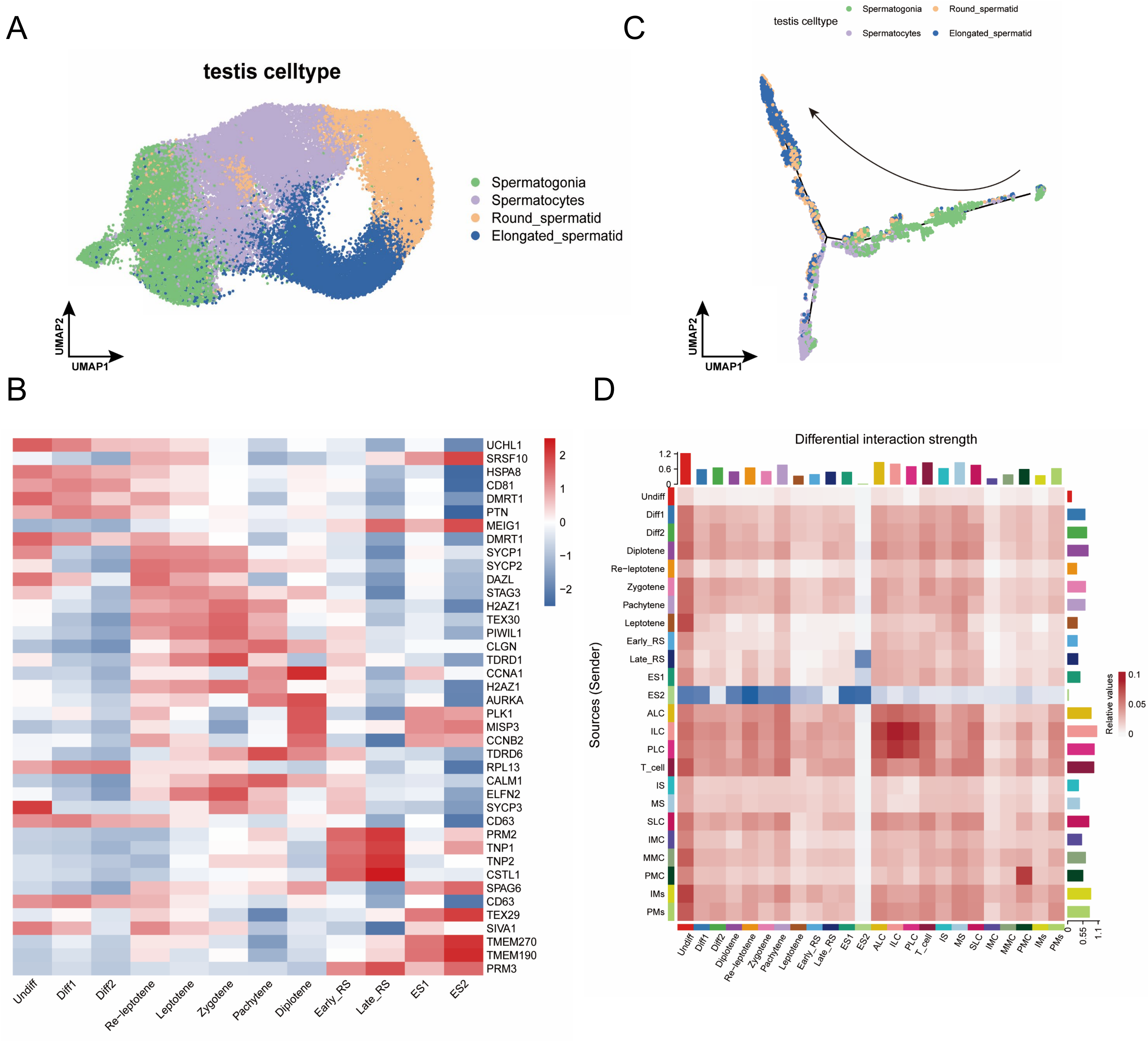
A
testis celltype
Spermatogonia
Spermatocytes
Round_spermatid
Elongated_spermatid
UMAP2
UMAP1
B
UCHL1
SRSF10
HSPA8
CD81
DMRT1
PTN
MEIG1
DMRT1
SYCP1
SYCP2
DAZL
STAG3
H2AZ1
TEX30
PIWIL1
CLGN
TDRD1
CCNA1
H2AZ1
AURKA
PLK1
MISP3
CCNB2
TDRD6
RPL13
CALM1
ELFN2
SYCP3
CD63
PRM2
TNP1
TNP2
CSTL1
SPAG6
CD63
TEX29
SIVA1
TMEM270
TMEM190
PRM3
2
1
0
−1
−2
Undiff
Diff1
Diff2
Re−leptotene
Leptotene
Zygotene
Pachytene
Diplotene
Early_RS
Late_RS
ES1
ES2
C
testis celltype
Spermatogonia
Spermatocytes
Round_spermatid
Elongated_spermatid
UMAP2
UMAP1
D
Differential interaction strength
1.2
0.6
0
Sources (Sender)
Undiff
Diff1
Diff2
Diplotene
Re−leptotene
Zygotene
Pachytene
Leptotene
Early_RS
Late_RS
ES1
ES2
ALC
ILC
PLC
T_cell
IS
MS
SLC
IMC
MMC
PMC
IMs
PMs
Relative values
0.1
0.05
0
0
0.55
1.1

Fig S2 Construction of a spatial transcriptional map of bovine testis tissue

A UMAP of bovine germ cell, stained differently according to spermatogonia, spermatocyte, round-spermatid, elongated-spermatid. Scale bar resolution: 0.4 μm. **B** The heatmap of germ cell subpopulations marker gene expression. **C** Pseudo-time sequence trajectory diagram of germ cell development by Monocle. **D** Comparison of the strength of intercellular communications between calf and adult bovine.

Figure S3

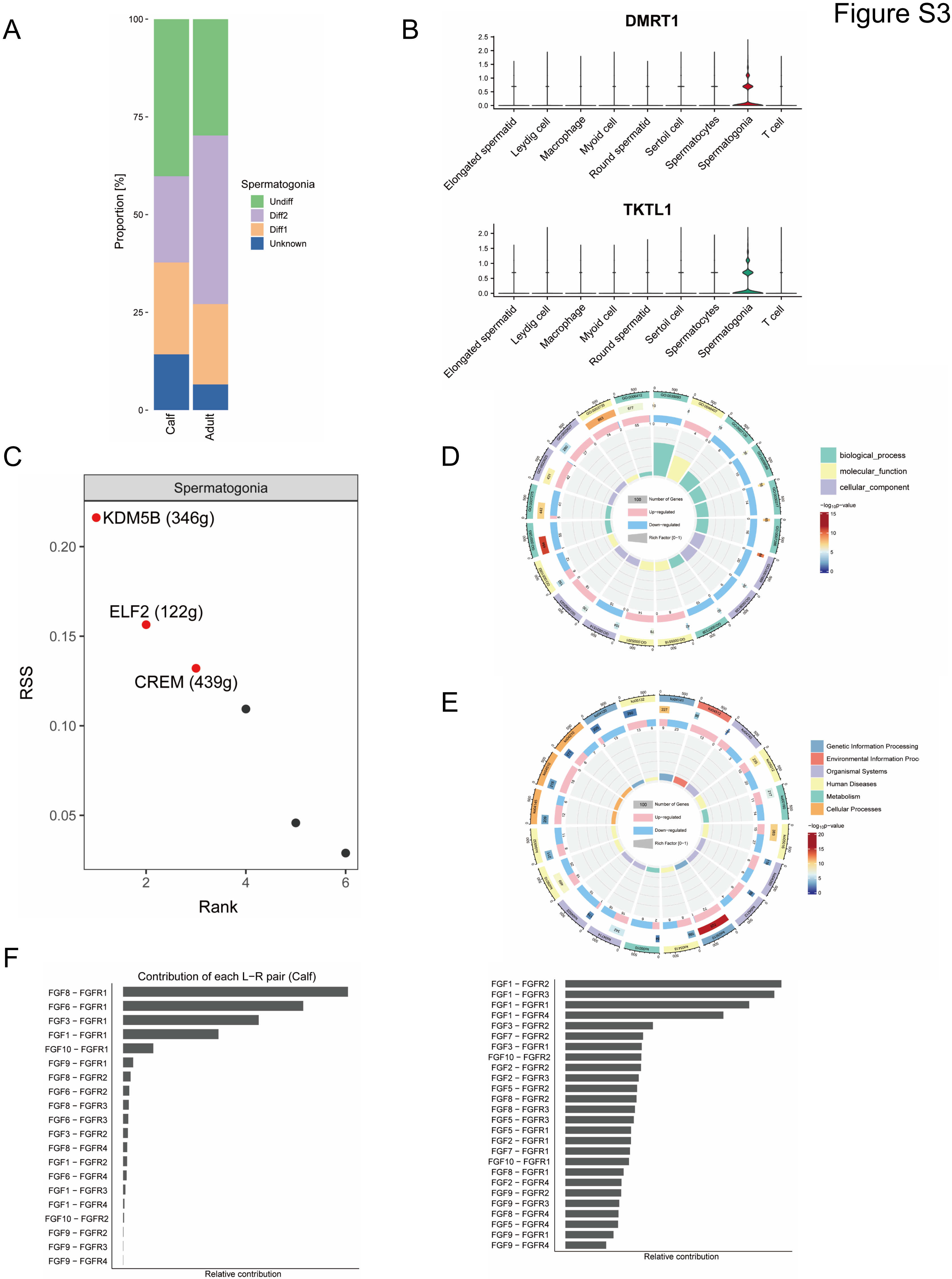
A
Proportion [%]
100
75
50
25
0
Calf
Adult
Spermatogonia
Undiff
Diff2
Diff1
Unknown
B
DMRT1
TKTL1
Elongated spermatid
Leydig cell
Macrophage
Myoid cell
Round spermatid
Sertoil cell
Spermatocytes
Spermatogonia
T cell
C
Spermatogonia
KDM5B (346g)
ELF2 (122g)
CREM (439g)
RSS
Rank
D
biological_process
molecular_function
cellular_component
E
Genetic Information Processing
Organismal Systems
Human Diseases
Metabolism
Cellular Processes
F
Contribution of each L−R pair (Calf)
FGF8 − FGFR1
FGF6 − FGFR1
FGF3 − FGFR1
FGF1 − FGFR1
FGF10 − FGFR1
FGF9 − FGFR1
FGF8 − FGFR2
FGF6 − FGFR2
FGF8 − FGFR3
FGF6 − FGFR3
FGF3 − FGFR2
FGF8 − FGFR4
FGF1 − FGFR2
FGF6 − FGFR4
FGF1 − FGFR3
FGF1 − FGFR4
FGF10 − FGFR2
FGF9 − FGFR2
FGF9 − FGFR3
FGF9 − FGFR4
Relative contribution
FGF1 − FGFR2
FGF1 − FGFR3
FGF1 − FGFR1
FGF1 − FGFR4
FGF3 − FGFR2
FGF7 − FGFR2
FGF3 − FGFR1
FGF10 − FGFR2
FGF2 − FGFR2
FGF2 − FGFR3
FGF5 − FGFR2
FGF8 − FGFR2
FGF8 − FGFR3
FGF5 − FGFR3
FGF5 − FGFR1
FGF2 − FGFR1
FGF7 − FGFR1
FGF10 − FGFR1
FGF8 − FGFR1
FGF2 − FGFR4
FGF9 − FGFR2
FGF9 − FGFR3
FGF8 − FGFR4
FGF5 − FGFR4
FGF9 − FGFR1
FGF9 − FGFR4
Relative contribution

Figure S3 Heterogeneity of developmental processes in bovine spermatogonia

A The proportion of the number of spermatogonia subsets in the two age groups. **B** Violin plot expression pattern of DMRT1, TKTL1. **C** Regulon specificity score (RSS) of spermatogonia. **D** Circular plot of Gene Ontology (GO) enrichment analysis for Adult vs Calf. **E** Circular plot of KEGG enrichment analysis for Adult vs Calf. **F** FGF signaling pathway L-R pairs.

Figure S4

A

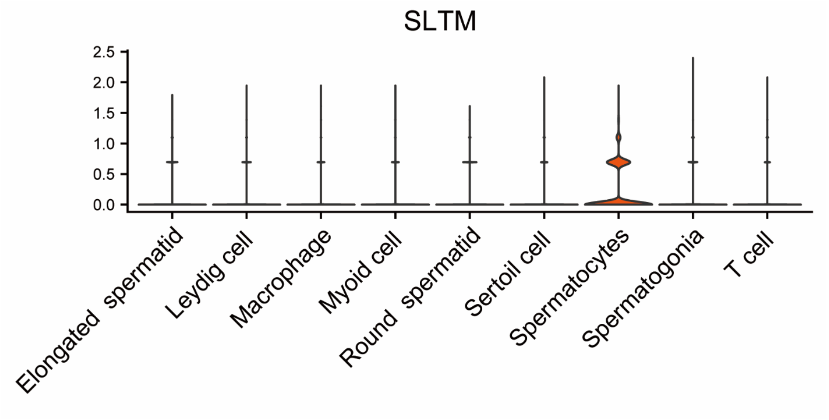

B

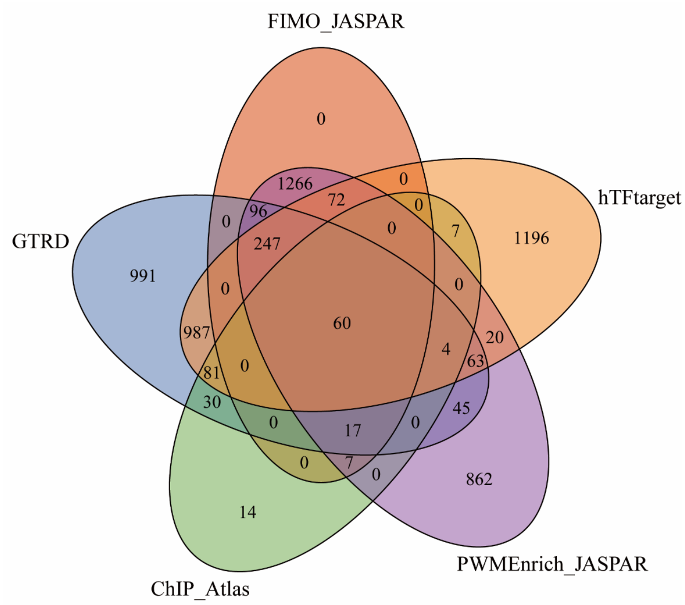

C

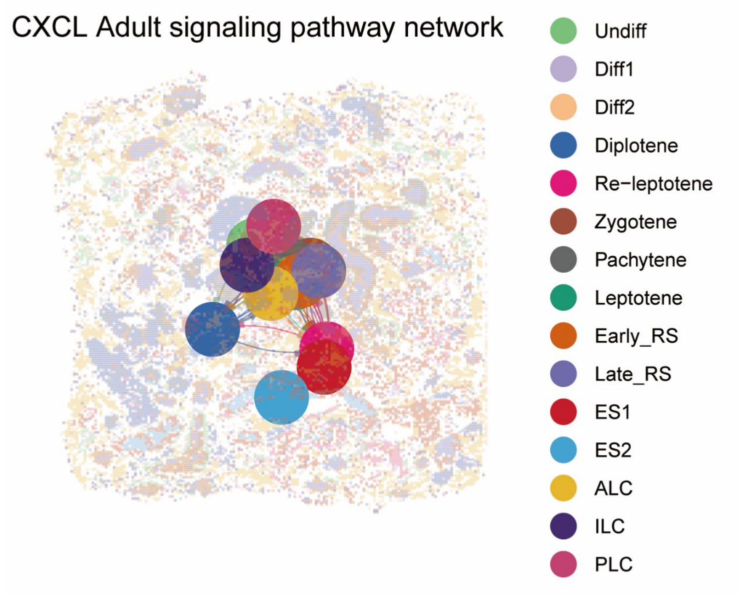

D

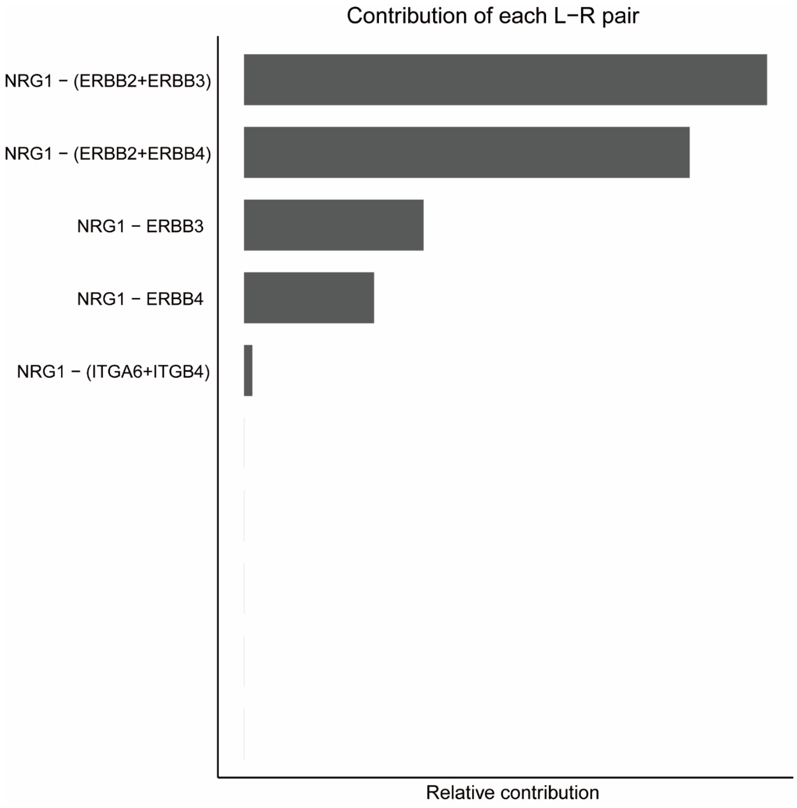

Figure S4 Characterization of spatial transcription in bovine spermatocyte revealed

A Violin plot expression pattern of SLTM. **B** Multi-platform prediction of RFX2 target genes. **C** Spatial distribution of CXCL signaling pathway in germ cell subtypes. **D** NRG signaling pathway L-R pairs.

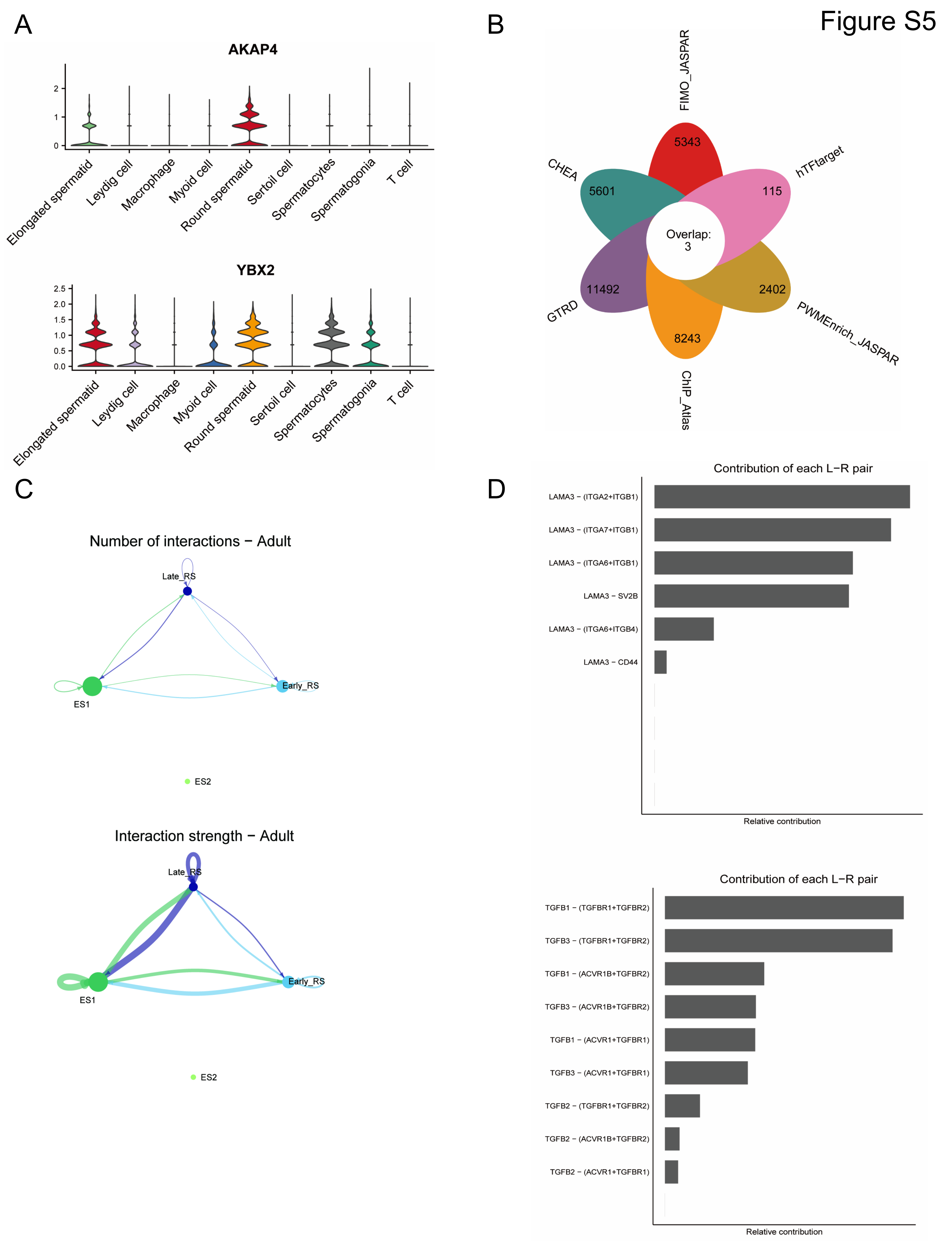
Figure S5
A
AKAP4
YBX2
Elongated spermatid
Leydig cell
Macrophage
Myoid cell
Round spermatid
Sertoil cell
Spermatocytes
Spermatogonia
T cell
B
FIMO_JASPAR
5343
hTFtarget
115
PWMEnrich_JASPAR
2402
ChIP_Atlas
8243
GTRD
11492
CHEA
5601
Overlap:
3
C
Number of interactions − Adult
Interaction strength − Adult
Late_RS
Early_RS
ES1
ES2
D
Contribution of each L−R pair
LAMA3 − (ITGA2+ITGB1)
LAMA3 − (ITGA7+ITGB1)
LAMA3 − (ITGA6+ITGB1)
LAMA3 − SV2B
LAMA3 − (ITGA6+ITGB4)
LAMA3 − CD44
Relative contribution
Contribution of each L−R pair
TGFB1 − (TGFBR1+TGFBR2)
TGFB3 − (TGFBR1+TGFBR2)
TGFB1 − (ACVR1B+TGFBR2)
TGFB3 − (ACVR1B+TGFBR2)
TGFB1 − (ACVR1+TGFBR1)
TGFB3 − (ACVR1+TGFBR1)
TGFB2 − (TGFBR1+TGFBR2)
TGFB2 − (ACVR1B+TGFBR2)
TGFB2 − (ACVR1+TGFBR1)
Relative contribution

Figure S5 Dynamic transcription expression patterns during bovine sperm deformation

A Violin plot expression pattern of YBX2, AKAP4. **B** Multi-platform prediction of CREM target genes. **C** The number and strength network diagram of interaction between spermatid. **D** LAMININ and TGFb signaling pathways L-R pairs.

Figure S6

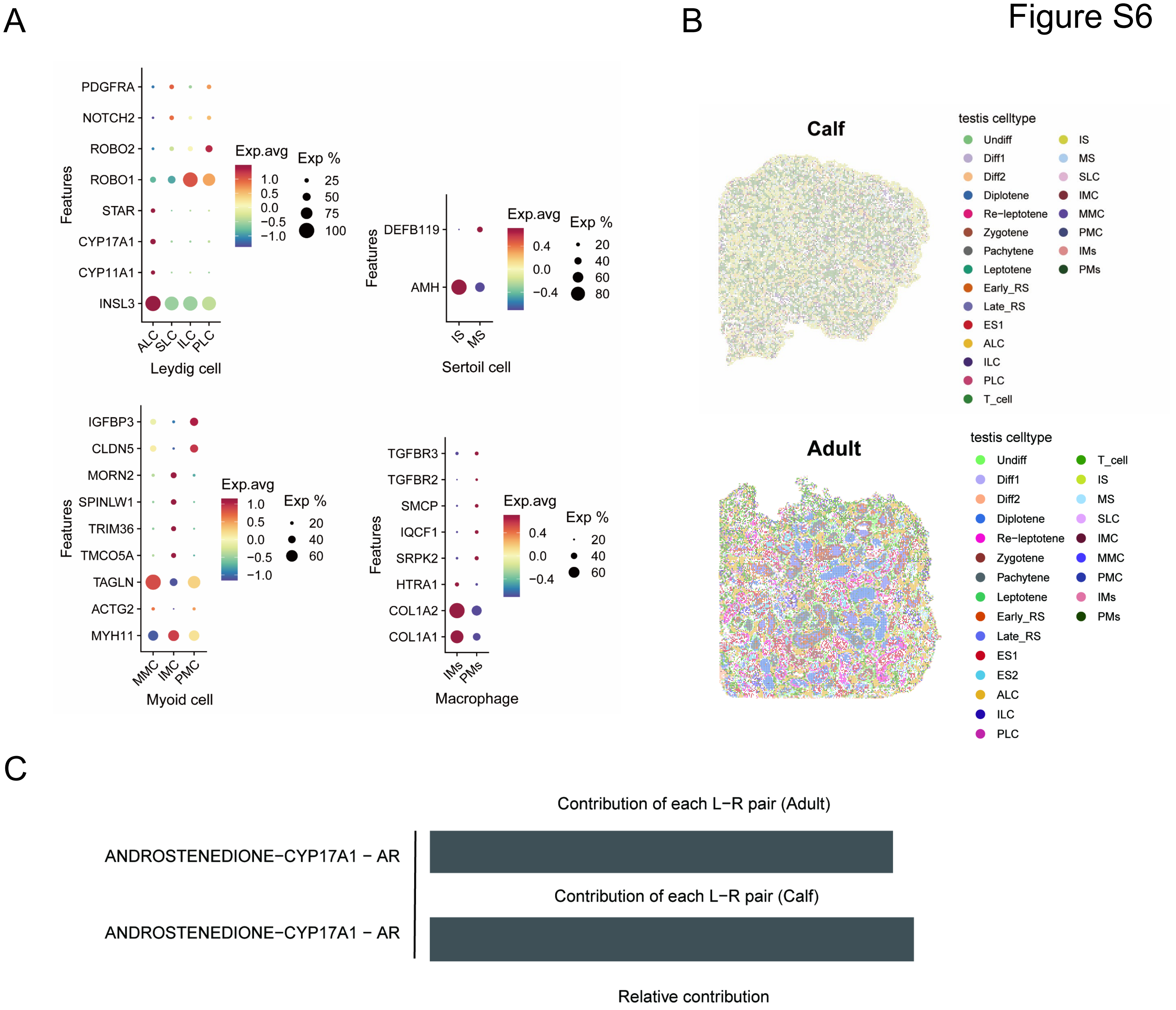
A
B
C
PDGFRA
NOTCH2
ROBO2
ROBO1
STAR
CYP17A1
CYP11A1
INSL3
Features
Exp.avg
1.0
0.5
0.0
−0.5
−1.0
Exp %
25
50
75
100
ALC
SLC
ILC
PLC
Leydig cell
DEFB119
AMH
Features
Exp.avg
0.4
0.0
−0.4
Exp %
20
40
60
80
IS
MS
Sertoil cell
IGFBP3
CLDN5
MORN2
SPINLW1
TRIM36
TMCO5A
TAGLN
ACTG2
MYH11
Features
Exp.avg
1.0
0.5
0.0
−0.5
−1.0
Exp %
20
40
60
MMC
IMC
PMC
Myoid cell
TGFBR3
TGFBR2
SMCP
IQCF1
SRPK2
HTRA1
COL1A2
COL1A1
Features
Exp.avg
0.4
0.0
−0.4
Exp %
20
40
60
IMs
PMs
Macrophage
Calf
testis celltype
Undiff
Diff1
Diff2
Diplotene
Re−leptotene
Zygotene
Pachytene
Leptotene
Early_RS
Late_RS
ES1
ALC
ILC
PLC
T_cell
IS
MS
SLC
IMC
MMC
PMC
IMs
PMs
Adult
testis celltype
Undiff
Diff1
Diff2
Diplotene
Re−leptotene
Zygotene
Pachytene
Leptotene
Early_RS
Late_RS
ES1
ES2
ALC
ILC
PLC
T_cell
IS
MS
SLC
IMC
MMC
PMC
IMs
PMs
Contribution of each L−R pair (Adult)
ANDROSTENEDIONE−CYP17A1 − AR
Contribution of each L−R pair (Calf)
ANDROSTENEDIONE−CYP17A1 − AR
Relative contribution

Figure S6 Spatial transcriptional characteristics of bovine testicular somatic cell subpopulations

A The dotplot heatmap of somatic cell subpopulations marker gene expression. **B** Spatial slice mapping of testicular cell type distribution included all subpopulations. **C** Androstenedione signaling pathway L-R pairs.
